# Phase 1, First‐In‐Human, Single‐/Multiple‐Ascending Dose Study of Iluzanebart in Healthy Volunteers

**DOI:** 10.1002/acn3.70033

**Published:** 2025-04-01

**Authors:** Andreas Meier, Spyros Papapetropoulos, Andrew Marsh, Kelly Neelon, David Stiles, Ryan O'Mara, Evan A. Thackaberry, Marco Colonna, Raj Rajagovindan

**Affiliations:** ^1^ Formerly Vigil Neuroscience, Inc. Watertown Massachusetts USA; ^2^ Vigil Neuroscience, Inc. Watertown Massachusetts USA; ^3^ Department of Pathology and Immunology Washington University School of Medicine St Louis Missouri USA

**Keywords:** adult‐onset leukoencephalopathy with axonal spheroids and pigmented glia, CSF‐1 receptor, iluzanebart, microglia, TREM2

## Abstract

**Objective:**

To evaluate the safety, tolerability, pharmacokinetics, and pharmacodynamics of iluzanebart, a fully human monoclonal antibody TREM2 (triggering receptor expressed on myeloid cells 2) agonist, after single‐ (SAD) and multiple‐ascending‐dose (MAD) administration.

**Methods:**

Healthy adult volunteers (*N* = 136) received intravenous placebo or iluzanebart 1–60 mg/kg (SAD) or 10–60 mg/kg (MAD) followed by serial pharmacokinetics and safety assessments. Safety assessments included adverse events (AEs), vital signs, electrocardiograms, and clinical laboratory evaluations. Pharmacokinetics were assessed through noncompartmental analysis. The study also included open‐label cohorts (3, 10, 20, 40, 60 mg/kg SAD; 10, 20, 40 mg/kg MAD) for cerebrospinal fluid (CSF) collection for exploratory pharmacodynamic biomarker analysis.

**Results:**

Iluzanebart was safe and well tolerated following single and multiple doses of up to 60 mg/kg. Most AEs were mild and resolved spontaneously. The most frequently reported AE was pruritus. No serious AEs or investigational product–related clinically meaningful changes in vital signs, electrocardiograms, or laboratory assessments were reported. Iluzanebart serum exposure was related to dose, with a 29‐day half‐life that is supportive of monthly dosing and confirmed central nervous system (CNS) exposure (≈0.15% CSF‐to‐serum ratio). Durable and dose‐dependent target engagement, evidenced by marked reductions in soluble TREM2 and increased soluble CSF1R (colony‐stimulating factor 1 receptor) and osteopontin/SPP1 (secreted phosphoprotein 1) levels in CSF, was observed, indicating that iluzanebart changes microglial activity following single and repeat dosing.

**Interpretation:**

Iluzanebart demonstrated favorable safety, tolerability, pharmacokinetics, and pharmacological activity in the CNS, supporting further clinical development for adult‐onset leukoencephalopathy with axonal spheroids and pigmented glia.

## 1 Introduction

Colony‐stimulating factor 1 receptor (CSF1R) adult‐onset leukoencephalopathy with axonal spheroids and pigmented glia (ALSP) is a rare, autosomal dominant, progressive, debilitating neurodegenerative disorder caused primarily by loss‐of‐function variants of the *CSF1R* gene [1, 2]. CSF1R is a transmembrane tyrosine kinase receptor for CSF1 and interleukin‐34 (IL‐34), two myeloid growth‐promoting cytokines [3]. Binding of CSF1 or IL‐34 to CSF1R enables receptor dimerization, autophosphorylation, activation of phosphatidylinositol 3‐kinase (PI3K), phospholipase C (PLC), and extracellular signal‐regulated kinase (ERK), and recruitment of Src tyrosine kinase [3, 4, 5]. Once recruited, Src phosphorylates the immune signaling adaptor protein DNAX‐activating protein of 12 kDa (DAP12), triggering spleen tyrosine kinase (SYK) and activation of additional downstream mediators [3, 5]. Activation of CSF1R promotes the survival, proliferation, differentiation, and function of microglia [1, 2, 6], the sentinel immune cells that interact with various components of the central nervous system (CNS) to maintain brain homeostasis and white matter integrity [7, 8]. In CSF1R‐ALSP, *CSF1R* variants result in impaired CSF1R signaling, leading to microglial loss and dysfunction [9].

Neuropathologic findings in CSF1R‐ALSP include demyelination of cerebral white matter, axonal swelling, and pigmented glial cells [1]. Clinically, CSF1R‐ALSP is characterized by a rapidly progressive decline in cognitive, neuropsychiatric, and motor functions, with typical symptom onset around 40–50 years of age (mean [median; range]: 43.2 [42; 18–86] years) and resulting in incapacitation within 3–4 years of disease onset and death within 6–8 years, on average [1, 10, 11, 12]. Based on the population‐scale whole‐exome sequencing data set of the UK BioBank, recent research has shown the rate of pathogenic and likely pathogenic variants of *CSF1R* to be 281 per million [13]. These data imply that the number of CSF1R‐ALSP cases documented by the literature may underestimate the prevalence of this disease, possibly due in part to its high frequency of initial misdiagnosis [1, 12, 14]. Patients with CSF1R‐ALSP have been known to be frequently misdiagnosed with more common neurological diseases such as multiple sclerosis and Alzheimer's disease [15, 16]. No disease‐modifying therapies are currently approved to treat CSF1R‐ALSP, highlighting a critical unmet need for therapies that slow the rapid progression of the disease [1].

Triggering receptor expressed on myeloid cells 2 (TREM2) is also a key microglial transmembrane receptor that plays an essential role in normal microglial function, supported by the observation that loss‐of‐function variants in the human *TREM2* gene result in Nasu‐Hakola disease, a rare leukodystrophy caused by lipid accumulation, demyelination, axonal loss, and gliosis and characterized by clinical symptoms that include bone cysts as well as cognitive decline, behavioral changes, motor symptoms, and seizures [8, 17, 18]. Stimulation of TREM2 by endogenous substrates, including myelin‐associated phospholipids, cellular debris, and lipoproteins (e.g., apolipoprotein E, high‐density lipoprotein, and low‐density lipoprotein), triggers the formation of a receptor complex between TREM2 and the adaptor DAP12, which contains an immunoreceptor tyrosine‐based activation motif (ITAM) [3, 18, 19]. This complexing promotes DAP12 phosphorylation and, in a pathway parallel to that initiated upon CSF1R activation, phosphorylated DAP12 recruits and activates SYK, orchestrating critical downstream pathways, including PI3K and PLCγ, to promote cellular proliferation and survival, regulate inflammation, and mediate phagocytosis [3]. Not only has positive modulation of the TREM2 transmembrane receptor been shown to enhance TREM2‐dependent microglial function [20], but TREM2 has also been shown to enhance CSF1R signaling through SYK activation [1, 3, 5, 21]. Thus, TREM2 and CSF1R in part share common downstream intracellular signaling and microglial functions in the CNS, play key roles in microglial survival, are both established modifiers of neurodegenerative disease based on human genetic studies, and converge on a shared intracellular signaling axis via SYK kinase [1]. This convergence of pathways provides a biological rationale for TREM2 agonism to compensate for CSF1R‐driven deficits and slow CSF1R‐ALSP disease progression [1].

Iluzanebart (formerly VGL101) is a fully human immunoglobulin G1 (IgG1) monoclonal antibody with reduced effector function designed to activate TREM2 to compensate for CSF1R dysfunction and restore the neuroprotective and homeostatic functions of microglia [22]. In an in vitro CSF1R‐ALSP model of induced haploinsufficiency in induced pluripotent stem cell–derived microglia, iluzanebart increased soluble CSF1R levels and rescued cells from the disease phenotype by reducing apoptosis and restoring viability and morphology [23]. Iluzanebart is in development for the potential treatment of CSF1R‐ALSP [24], and a phase 2 trial in individuals with CSF1R‐ALSP is underway (NCT05677659) [25].

Here, we present phase 1 data on the safety, tolerability, pharmacokinetics (PK), and pharmacodynamics (PD) following single‐ (SAD) and multiple‐ascending dosing (MAD) of intravenous (IV) iluzanebart in healthy adult volunteers (HVs).

## 2 Methods

### 2.1 Study Design and Participants

**FIGURE 1:**
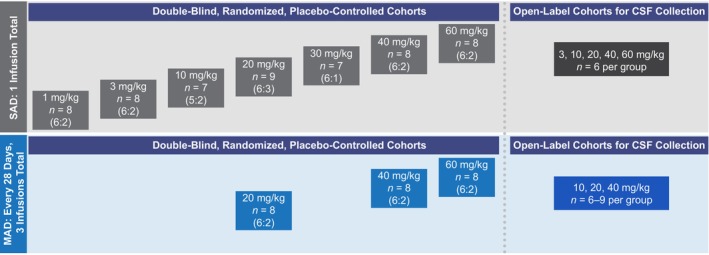
Study design. CSF, cerebrospinal fluid; MAD, multiple‐ascending dose; SAD, single‐ascending dose.

This study is a two‐part, first‐in‐human, phase 1, randomized, double‐blind, placebo‐controlled SAD and MAD trial of IV iluzanebart in HVs aged 18 to 55 years (Figure 1) conducted at three sites in the United States and Australia (Australian New Zealand Clinical Trials Registry [anzctr.org.au]: ACTRN12622000933752). The SAD and MAD parts each included an open‐label cohort for cerebrospinal fluid (CSF) collection to characterize PK and exploratory PD biomarkers in CSF. Eligible HVs were identified based on medical history, physical examination, vital signs, 12‐lead electrocardiogram (ECG), and clinical laboratory evaluations. A body mass index between 18.5 and 32.0 kg/m^2^ was required, and volunteers had to be nonsmokers (per US enrollment criteria) or occasional smokers (i.e., not smoking every day; per Australian enrollment criteria). Full inclusion and exclusion criteria are listed in the Supporting Information.

In each double‐blind cohort, eight HVs were planned to be randomized in a 6:2 ratio to receive a single dose of IV placebo or IV iluzanebart (1, 3, 10, 20, 30, 40, or 60 mg/kg) administered over 60 min (SAD; *N* = 56, as planned) or 3 doses of IV placebo or iluzanebart (20, 40, or 60 mg/kg) administered once every 28 days for a total of 3 administrations (MAD; *N* = 24, as planned). Randomization was carried out according to randomization codes generated by Parexel International (Glendale, CA, USA). SAD cohorts were subject to sentinel dosing, during which the first two HVs in each cohort were randomized and administered iluzanebart (*n* = 1) or placebo (*n* = 1). The remaining six HVs in each cohort were randomized in a 5:1 ratio to iluzanebart or placebo and received study treatment only after investigator review of safety data up to 72 h post dose for the first two HVs. For SAD, the remaining HVs in each cohort were dosed in a staggered fashion such that, after the sentinel pair, no two participants were dosed simultaneously. In all cohorts, dose escalation was performed sequentially, with available PK data and at least 7 days of safety data reviewed prior to escalation to the next dose cohort; safety review for dose escalation occurred after at least six HVs reached at least 7 days after final dosing. HVs who discontinued the study prematurely could be replaced at the discretion of the sponsor. For CSF collection and analyses, HVs were also assigned to separate open‐label cohorts for a single dose of IV iluzanebart 3, 10, 20, 40, or 60 mg/kg (SAD; *n* = 6 per group, as planned) or for 3 doses of IV iluzanebart 10, 20, or 40 mg/kg administered once every 28 days for a total of 3 administrations (MAD; *n* = 6–9 per group, as planned); these open‐label cohorts were not subject to sentinel dosing.

The total SAD study duration for each HV was approximately 113 days, including up to 28 days for screening, admission, and baseline assessments, a 1‐day treatment period, and an 84‐day follow‐up period. For MAD, the total study duration for each HV was approximately 169 days, including up to 28 days for screening, admission, and baseline assessments, a 57‐day treatment period, and a follow‐up period of 84 days after the last dosing visit (day 57) up to the end of study (EOS) visit.

Protocol‐specified study‐stopping criteria stipulated discontinuation of the study if any of the following occurred: development of the same category grade 3 (severe) adverse event (AE) related to the study drug in two HVs, development of a grade 4 (potentially life‐threatening) AE related to the study drug in any HV, or death of an HV at any time in relation to the study drug. The study was conducted in compliance with Good Clinical Practice, the Declaration of Helsinki, and all national and local legal requirements. The protocol and all amendments were reviewed and approved by an institutional review board at each participating investigative site prior to study initiation at the respective site, and all HVs provided written informed consent prior to the initiation of any study procedures.

### 2.2 Sample Collection and Analysis

Blood samples (serum) for PK analyses after SAD were collected at 0 (predose on day 1), 1, 2, 4, 8, 24, 36, and 48 h (± 15 min) and days 8, 15, 29, 47, and 85/EOS (all ± 1 day) after dosing. For MAD, blood samples for PK and PD analyses were collected at: 0 (predose on day 1), 1, 2, 4, 8, 24, and 48 h (± 15 min) and days 8 and 15 (± 1 day) after dose 1; 0 (predose), 1, and 4 h (± 15 min) after dose 2 (on day 29); and 0 (predose on day 1), 1, 2, 4, 8, 24, and 48 h (± 15 min) and days 7, 14, 28, 46, and 84 after dose 3 (on day 57; corresponding to study days 64, 71, 85, 103, and 141). Blood samples for immunogenicity analyses were collected predose on day 1 and at days 29 and 85 after dosing for SAD and 0 h (predose) before doses 1, 2, and 3 as well as day 85 after dose 3 (corresponding to day 141) for MAD. CSF was collected by lumbar puncture on day −2 (predose baseline), day 3 (48 h post dosing), and day 15 (336 h [± 1 day] post dosing) for SAD and on day −2 (predose baseline), day 59 (48 h post last dosing), and day 85 (672 h [± 1 day] post last dosing) for MAD.

For biomarker analysis, approximately 8 mL of CSF was collected in a 15 mL polypropylene canonical tube, split into aliquots of 250 μL in 2 mL cryovials, placed on dry ice, and stored at −80°C. Frozen samples were shipped to Immunologix Laboratories (Tampa, FL, USA). CSF samples for biomarkers were processed and analyzed using validated assays for soluble CSF1R (sCSF1R), soluble TREM2 (sTREM2), and osteopontin/secreted phosphoprotein 1 (SPP1) (Table S1). Concentrations of sCSF1R and sTREM2 in CSF were assayed using commercial enzyme‐linked immunosorbent assay kits (R&D Systems, Minneapolis, MN, USA). Samples were diluted to a minimum required dilution of 1/5 (sTREM2) and 1/64 (sCSF1R) with reagent diluent. Absorbance was read using a SpectraMax Plus 384 Microplate Reader (Molecular Devices, San Jose, CA, USA), with the wavelength set to 450 nm (detection) and 570 nm (background). Relative protein concentrations were calculated from their optical density in Watson LIMS Software (Thermo Fisher Scientific, Waltham, MA, USA) using the standard curve. Osteopontin/SPP1 concentration was assayed on the Ella microfluidic platform (Bio‐Techne, Minneapolis, MN, USA) using a commercial immunoassay kit (Ella Simple Plex Human Osteopontin 2nd Gen Cartridge; ProteinSimple, San Jose, CA, USA) following the manufacturer's instructions. Samples were diluted to a minimum required dilution of 1/100 with sample diluent. Relative fluorescence units were obtained from the Simple Plex Explorer software (Bio‐Techne). Relative protein concentrations were calculated from the relative fluorescence in Watson LIMS Software using the lot‐specific factory calibration curve.

The presence of antidrug antibodies (ADAs) in serum was determined using a validated affinity capture elution format assay (Charles River Laboratories, Mattawan, MI, USA). A screening assay was performed, followed by a confirmatory assay for any positive samples. Samples that were positive in the confirmatory assay were then titered and reported as positive for ADAs.

### 2.3 Endpoints

The primary endpoints were the safety and tolerability of iluzanebart as assessed by AEs, clinical laboratory tests, ECGs, and vital sign measurements. Infusion reactions were assessed as AEs of special interest and were graded as 1 (mild), 2 (moderate), 3 (severe), or 4 (potentially life‐threatening). Secondary endpoints included single‐dose and multiple‐dose serum PK parameters (e.g., area under the concentration‐time curve from predose [time 0] to the time of the last quantifiable concentration [AUC_0‐last_], extrapolated to infinite time [AUC_0‐inf_], or over a dosing interval [AUC_0‐τ_]; maximum serum concentration [*C*_max_]; time to *C*_max_ [*T*_max_]; terminal elimination half‐life [t_1/2_]; total body clearance [CL]; volume of distribution at steady state [Vss]; mean residence time [MRT]; concentration prior to dose administration [*C*_trough_]; accumulation ratio [Ro*C*_max_; *C*_max_ at last dose divided by *C*_max_ at day 1]; and accumulation ratio [RoAUC_0‐τ_; AUC_0‐τ_ at last dose divided by AUC_0‐τ_ at first dose]) and characterization of ADA levels following single‐ and multiple‐dose administration of iluzanebart. Exploratory endpoints included iluzanebart CSF exposure and changes from baseline in PD biomarkers in CSF.

CSF biomarkers included sTREM2 (the product of proteolytic cleavage of TREM2 on the cell surface and a proximal biomarker of TREM2 engagement [26]), sCSF1R (a downstream biomarker of microglial activity [26]), and osteopontin/SPP1 (a downstream biomarker associated with neuroprotective microglia) [27, 28].

### 2.4 Statistical Analysis

Sample sizes were selected on the basis of clinical judgment and not on formal statistical power analysis. The number of HVs selected was based on feasibility and was considered sufficient to meet the study objectives.

All demographic, safety, and PK and PD data were summarized (by treatment group [iluzanebart or placebo] for demographic and safety data and by cohort for PK and PD data) using descriptive statistics. Additionally, for each PD analyte, percentage changes from baseline (defined as the last measurement taken prior to dosing) at each post‐dose time point and for each dose level and cohort were assessed using a two‐sided paired *t* test (*α* = 0.05) of the null hypotheses that the treatment group mean change was equal to zero.

Demographic and safety data were summarized for all randomized HVs who received at least one dose of the study drug (defined as the safety population). PK analyses were performed in the PK population, which included all HVs in the safety population with sufficient serum concentration data available to facilitate the calculation of at least one PK parameter and no important protocol violations affecting PK variables. PD analyses were conducted in the PD population, consisting of all HVs in the safety population with sufficient CSF concentration data available to calculate at least one PD parameter and no important protocol violations affecting PD analysis. The numbers and percentages of AEs were tabulated by system organ class and preferred term according to the Medical Dictionary for Regulatory Activities version 26.0. All AEs were categorized according to intensity and relationship to treatment. Missing PK and PD data were not imputed.

Statistical analyses were performed using SAS (SAS Institute Inc., Cary, NC, USA) version 9.4 or higher. Single‐ and multiple‐dose PK parameters were derived from the serum concentration‐time curves using actual sampling times and standard noncompartmental analysis methods with Phoenix WinNonlin (Certara Inc., Mountain View, CA, USA) version 8.2 or higher.

## 3 Results

### 3.1 Participant Demographics and Disposition

The study enrolled a total of 135 HVs. The numbers of HVs per cohort are described in Figure 1, and demographic characteristics are shown in Table 1. Overall, mean (standard deviation) ages were 35.2 (9.6) years (iluzanebart, *n* = 116) and 38.1 (9.3) years (placebo, *n* = 19), and most HVs were male (57.0%), White (68.9%), and not Hispanic or Latino (63.0%).

**TABLE 1:** Demographic characteristics (safety population).

Characteristic	SAD cohorts		MAD cohorts		All HVs	
	Iluzanebart (*n* = 73)	Placebo (*n* = 13)	Iluzanebart (*n* = 43)	Placebo (*n* = 6)	Iluzanebart (*n* = 116)	Placebo (*n* = 19)
Age, mean (SD), years	36.2 (9.9)	37.4 (10.0)	33.5 (9.0)	39.5 (8.3)	35.2 (9.6)	38.1 (9.3)
Sex, *n* (%)						
Female	29 (39.7)	8 (61.5)	18 (41.9)	3 (50.0)	47 (40.5)	11 (57.9)
Male	44 (60.3)	5 (38.5)	25 (58.1)	3 (50.0)	69 (59.5)	8 (42.1)
Race, *n* (%)						
American Indian or Alaska Native	2 (2.7)	0	1 (2.3)	0	3 (2.6)	0
Asian	6 (8.2)	1 (7.7)	5 (11.6)	0	11 (9.5)	1 (5.3)
Black or African American	14 (19.2)	2 (15.4)	7 (16.3)	0	21 (18.1)	2 (10.5)
Native Hawaiian or other Pacific Islander	1 (1.4)	0	0	0	1 (0.9)	0
White	48 (65.8)	9 (69.2)	30 (69.8)	6 (100)	78 (67.2)	15 (78.9)
Other^a^	2 (2.7)	1 (7.7)	0	0	2 (1.7)	1 (5.3)
Ethnicity, *n* (%)						
Hispanic or Latino	25 (34.2)	4 (30.8)	18 (41.9)	0	43 (37.1)	4 (21.1)
Not Hispanic or Latino	47 (64.4)	9 (69.2)	23 (53.5)	6 (100)	70 (60.3)	15 (78.9)
Not reported or unknown	1 (1.4)	0	2 (4.7)	0	3 (2.6)	0
BMI, mean (SD), kg/m^2^	25.6 (2.9)	25.5 (2.7)	25.7 (2.6)	25.9 (3.1)	25.7 (2.7)	25.6 (2.7)

Abbreviations: BMI, body mass index; HV, healthy volunteer; MAD, multiple‐ascending dose; SAD, single‐ascending dose; SD, standard deviation.

^a^ Participants self‐identified by checking “Other” category.

**FIGURE 2:**
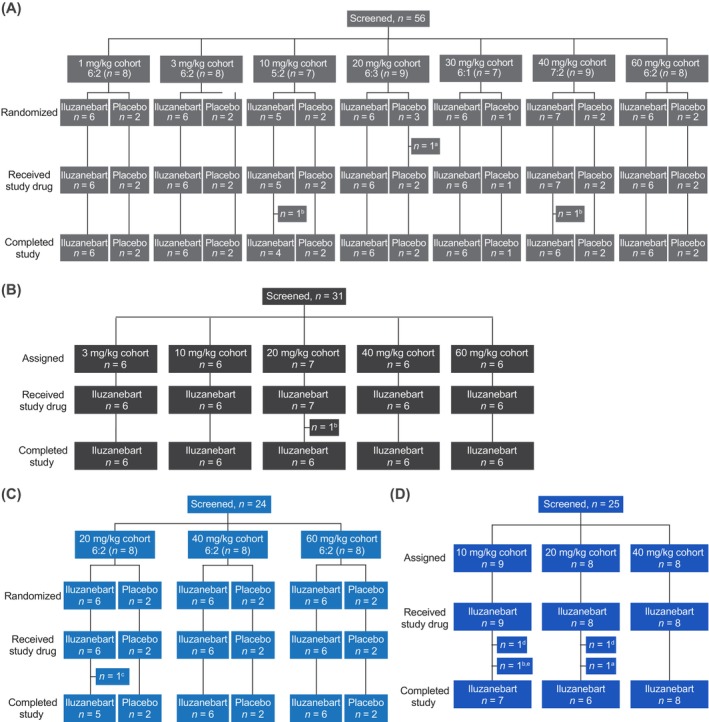
CONSORT diagrams for (A) SAD double‐blind, randomized cohorts, (B) SAD open‐label cohorts for CSF collection, (C) MAD double‐blind, randomized cohorts, and (D) MAD open‐label cohorts for CSF collection. Discontinued study due to ^a^participant withdrawal, ^b^adverse event, ^c^loss to follow‐up, or ^d^protocol deviation. ^e^Participant withdrawn from study on day 2 (after first dosing) due to an adverse event that had occurred on day −1 (before first dosing). CONSORT, Consolidated Standards of Reporting Trials; CSF, cerebrospinal fluid; MAD, multiple‐ascending dose; SAD, single‐ascending dose.

Of 136 individuals screened and randomized or assigned to study drug, one HV withdrew prior to dosing and 135 HVs received iluzanebart or placebo (Figure 2). Of these, 83 of 86 HVs (96.5%) in the SAD cohorts and 44 of 49 HVs (89.8%) in the MAD cohorts completed the study. The eight HVs who received the study drug but discontinued the study prematurely did so due to AEs (*n* = 4; two were considered related to investigational product [see below]), protocol violations (*n* = 2), withdrawal of consent (*n* = 1), and loss to follow‐up (*n* = 1) (Figure 2).

### 3.2 Safety and Tolerability

Of the 116 and 19 HVs administered iluzanebart and placebo, respectively, 65 (56.0%) and six (31.6%) reported at least one AE (Table 2). Most AEs were mild and most resolved spontaneously. At least one infusion‐related reaction (including pruritus, throat tightness, throat irritation, paresthesia, oropharyngeal discomfort, dyspnea, or flushing) was reported by 25 of 116 HVs (21.6%). The most frequently reported investigational product–related AE was pruritus, which occurred in 11 of 73 HVs (15.1%) in the SAD cohorts (10 mg/kg, *n* = 2; 20 mg/kg, *n* = 2; 30 mg/kg, *n* = 1; 40 mg/kg, *n* = 3; 60 mg/kg, *n* = 3) and six of 43 HVs (14.0%) in the MAD cohorts (20 mg/kg, *n* = 3; 40 mg/kg, *n* = 1; 60 mg/kg, *n* = 2); all AEs of pruritus were mild in severity. Of the four HVs who discontinued the study due to AEs, one (10 mg/kg SAD) did so due to mild related throat tightness and pruritus, one (20 mg/kg SAD) due to mild unrelated COVID‐19 infection, one (40 mg/kg SAD) due to mild related paresthesia, and one (10 mg/kg MAD) discontinued the study on day 2 due to a moderate, nonserious, unrelated event of presyncope that had occurred the day before the first dosing. All four HVs recovered, and all of these AEs resolved. Overall, no serious AEs or severe investigational product–related AEs were reported in any of the SAD or MAD cohorts, and changes in vital signs, ECGs, and laboratory parameters were unremarkable.

**TABLE 2:** Summary of safety outcomes during the study (safety population).

Number of participants (%)	SAD cohorts		MAD cohorts		All HVs	
	Iluzanebart (*n* = 73)	Placebo (*n* = 13)	Iluzanebart (*n* = 43)	Placebo (*n* = 6)	Iluzanebart (*n* = 116)	Placebo (*n* = 19)
Any AE^a^	42 (57.5)	4 (30.8)	23 (53.5)	2 (33.3)	65 (56.0)	6 (31.6)
Mild	37 (50.7)	4 (30.8)	13 (30.2)	2 (33.3)	50 (43.1)	6 (31.6)
Moderate	4 (5.5)	0	6 (14.0)	0	10 (8.6)	0
Severe^b^	1 (1.4)	0	4 (9.3)	0	5 (4.3)	0
Serious AE	0	0	0	0	0	0
Study discontinuation due to AE^c^	3 (4.1)	0	0	0	3 (2.6)	0
Most common AEs^d^						
Pruritus^e^	12 (16.4)	0	6 (14.0)	0	18 (15.5)	0
Headache	10 (13.7)	1 (7.7)	5 (11.6)	0	15 (12.9)	1 (5.3)
Back pain	7 (9.6)	0	1 (2.3)	0	8 (6.9)	0
Paresthesia^e^	2 (2.7)	0	2 (4.7)	1 (16.7)	4 (3.4)	1 (5.3)
Throat irritation^e^	2 (2.7)	0	2 (4.7)	0	4 (3.4)	0
Presyncope	1 (1.4)	0	3 (7.0)	0	4 (3.4)	0
Procedural pain	2 (2.7)	0	2 (4.7)	0	4 (3.4)	0
COVID‐19	3 (4.1)	0	0	0	3 (2.6)	0
Throat tightness^e^	3 (4.1)	0	0	0	3 (2.6)	0
Dermatitis contact	1 (1.4)	0	2 (4.7)	0	3 (2.6)	0
Post–lumbar puncture syndrome	0	0	3 (7.0)	0	3 (2.6)	0
Any treatment‐related AE^a^, ^f^	25 (34.2)	1 (7.7)	16 (37.2)	1 (16.7)	41 (35.3)	2 (10.5)
Mild	24 (32.9)	1 (7.7)	13 (30.2)	1 (16.7)	37 (31.9)	2 (10.5)
Moderate	1 (1.4)	0	3 (7.0)	0	4 (3.4)	0
Severe	0	0	0	0	0	0
Study discontinuation due to treatment‐related AE^f^	2 (2.7)	0	0	0	2 (1.7)	0
Most common treatment‐related AEs^d^, ^f^						
Pruritus^e^	11 (15.1)	0	6 (14.0)	0	17 (14.7)	0
Headache	6 (8.2)	0	2 (4.7)	0	8 (6.9)	0
Throat irritation^e^	2 (2.7)	0	2 (4.7)	0	4 (3.4)	0
Throat tightness^e^	3 (4.1)	0	0	0	3 (2.6)	0

Abbreviations: AE, adverse event; HV, healthy volunteer; MAD, multiple‐ascending dose; SAD, single‐ascending dose.

^a^ For categorization by severity, participants who experienced > 1 AE were categorized only once based on the most severe AE that occurred.

^b^ One participant in the SAD 40 mg/kg open‐label cohort experienced an unrelated, nonserious, severe AE of back pain 14 days post dose; 1 participant in the MAD 20 mg/kg open‐label cohort experienced an unrelated, nonserious, severe AE of blood creatine phosphokinase increased 27 days after their second dose; 2 participants in the MAD 40 mg/kg open‐label cohort each experienced an unrelated, nonserious, severe AE of presyncope 2 days after their third dose; and 1 participant in the MAD 40 mg/kg open‐label cohort experienced an unrelated, nonserious, severe AE of post–lumbar puncture syndrome 2 days after their third dose. All 5 participants recovered without changes in study treatment, and all 5 AEs resolved.

^c^ No participants from the MAD cohorts experienced an AE after the first dose that led to study discontinuation; however, 1 participant in the MAD 10 mg/kg open‐label cohort was withdrawn from the study on day 2 (after first dosing) due to a moderate, nonserious AE of presyncope that had occurred on day −1 (before first dosing) that the investigator considered unrelated to the study product.

^d^ AEs that occurred in ≥ 3 participants in any group.

^e^ Pruritus, throat tightness, throat irritation, paresthesia, oropharyngeal discomfort, flushing, and dyspnea were classified as infusion‐related reactions.

^f^ Treatment‐related AEs were the events determined by the investigator to be “possibly related,” “probably related,” or “related” to the investigational study drug.

### 3.3 Immunogenicity

All HVs were ADA negative at each postbaseline visit during the MAD part of the study. These data demonstrate a lack of treatment‐emergent ADAs upon multiple dosing with iluzanebart.

### 3.4 Pharmacokinetics

**FIGURE 3:**
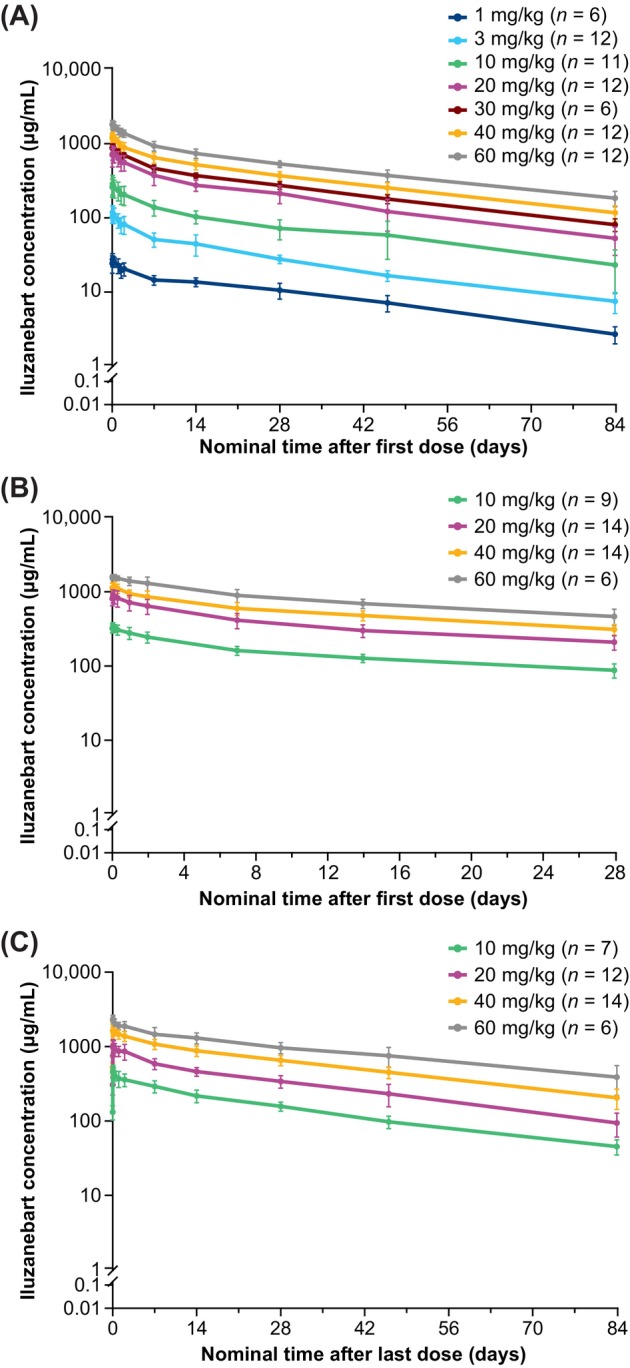
Iluzanebart pharmacokinetics (PK population): (A) SAD on day 1, (B) MAD^a^ on day 1, and (C) MAD^a^ on day 57.^b^ Data reported as mean ± SD. The early time points for each panel shown here (i.e., Days 1–2) are presented in Figure S3. ^a^Three administrations at 28‐day intervals. ^b^At time zero, 1 HV in the 3 mg/kg SAD cohort and 2 HVs in the 40 mg/kg MAD cohort had non‐zero concentration values (0.123–1.19 μg/mL), nominally above the quantification threshold of the assay (0.070 μg/mL) and likely due to inherent assay variability, whereas all concentrations at time zero in all other HVs were below the limit of quantification. The 3 non‐zero values at time zero have therefore been excluded from the dataset as outliers. HV, healthy volunteer; MAD, multiple‐ascending dose; PK, pharmacokinetic(s); SAD, single‐ascending dose; SD, standard deviation.

Iluzanebart displayed well‐characterized linear PK after single doses (Figure 3A), with mean serum AUC_0‐672_, AUC_0‐last_, AUC_0‐inf_, and *C*_max_ increasing proportionally with iluzanebart dose level from 1 to 60 mg/kg (Table 3). The observed half‐life of iluzanebart was approximately 29 days. Total body clearance, Vss, and Vz values were comparable between dose levels.

**TABLE 3:** Summary of PK parameters following a single IV infusion of iluzanebart.

Serum PK parameters^a^	Iluzanebart dose level						
	1 mg/kg (*n* = 6)	3 mg/kg (*n* = 12)	10 mg/kg (*n* = 11)	20 mg/kg (*n* = 12)	30 mg/kg (*n* = 6)	40 mg/kg (*n* = 12)	60 mg/kg (*n* = 12)
AUC_0‐inf_, h × μg/mL	20,420 (17.7)	59,730 (16.8)^b^	144,600 (31.6)^c^	424,200 (18.8)^d^	583,200 (12.7)	803,400 (16.7)^b^	1,170,000 (10.6)^e^
*C*_max_, μg/mL	27.70 (20.0)	121.9 (22.1)	290.2 (27.9)	794.4 (19.9)	937.7 (7.3)	1298 (12.1)	1880 (11.5)
*T*_max_, h^f^	2.01 (1.44, 4.01)	2.00 (1.03, 8.00)	2.00 (2.00, 7.93)	3.03 (1.00, 8.00)	2.00 (1.17, 3.70)	2.00 (1.17, 4.00)	2.00 (1.17, 8.02)
t_1/2_, h	709.5 (15.9)	680.6 (16.0)^b^	611.2 (23.6)^c^	640.5 (17.5)^d^	757.2 (8.9)	764.4 (10.5)^b^	778.8 (10.8)^e^

Abbreviations: AUC_0‐inf_, area under the serum concentration curve from predose (time 0) extrapolated to infinite time; *C*_max_, maximum serum concentration; CV, coefficient of variation; IV, intravenous; PK, pharmacokinetic(s); t_1/2_, terminal elimination half‐life; *T*_max_, time to *C*_max_.

^a^ Data reported as arithmetic mean (arithmetic mean CV %) unless otherwise indicated.

^b^ *n* = 10.

^c^ *n* = 7.

^d^ *n* = 11.

^e^ *n* = 8.

^f^ Data reported as median (min, max).

After repeat dosing, PK was predictable, with low variability (Figure 3B). AUC_0‐τ_ values (Table 4) following the first iluzanebart infusion (day 1) were comparable by dose level to those observed in the respective SAD cohorts. Following the third IV infusion (day 57), AUC_0‐τ_ increased with iluzanebart dose level. Maximum serum concentration occurred between 1.17 and 4.00 h post dose, with values ranging between 474.6 and 2318 μg/mL. Accumulation ratios between day 1 and day 57 were within the ranges of 1.4–1.8 (AUC_0‐τ_) and 1.2–1.4 (*C*_max_). Mean serum iluzanebart steady‐state clearance and Vz values were also comparable between dose levels. Iluzanebart PK did not appear to reach steady state by day 85 after the third IV infusion at any dose. The brain penetration of iluzanebart across all dose levels was approximately 0.15% CSF‐to‐serum ratio.

**TABLE 4:** Summary of PK parameters following the first and third IV infusions of iluzanebart administered once every 28 days.

Visit	Serum PK parameters^a^	Iluzanebart dose level			
		10 mg/kg (*n* = 9)	20 mg/kg (*n* = 14)	40 mg/kg (*n* = 14)	60 mg/kg (*n* = 6)
Day 1	AUC_0‐__τ_, h × μg/mL	96,530 (14.5)^b^	240,600 (19.4)	354,900 (16.1)	521,900 (16.8)
	*C*_max_, μg/mL	340.3 (14.2)^b^	909.8 (20.5)	1201 (16.0)	1647 (6.4)
	*T*_max_, h^c^	2.00 (1.00, 8.00)^b^	2.04 (1.00, 23.88)	4.00 (1.17, 24.27)	3.02 (1.20, 8.08)
Day 57	AUC_0‐τ_, h × μg/mL	161,000 (18.8)^d^	349,200 (15.1)^e^	636,600 (14.0)	900,200 (15.6)
	*C*_max_, μg/mL	474.6 (19.0)^d^	1095 (22.0)^e^	1673 (9.1)	2318 (13.4)
	*T*_max_, h^c^	2.00 (1.00, 2.00)^d^	4.00 (1.00, 48.01)^e^	1.25 (1.00, 4.17)	1.17 (1.17, 2.00)
	RoAUC_0‐τ_	1.662 (8.6)^d^	1.424 (9.1)^e^	1.810 (12.2)	1.730 (6.1)
	AR	1.410 (9.7)^d^	1.200 (20.9)^e^	1.413 (12.4)	1.405 (10.0)

Abbreviations: AR, accumulation ratio for *C*_max_; AUC_0‐τ_, area under the concentration‐time curve over the dosing interval; *C*_max_, maximum serum concentration; CV, coefficient of variation; IV, intravenous; PK, pharmacokinetic(s); Ro, accumulation ratio; *T*_max_, time to *C*_max_.

^a^ Data reported as arithmetic mean (arithmetic mean CV %) unless otherwise indicated.

^b^ *n* = 8.

^c^ Data reported as median (min, max).

^d^ *n* = 7.

^e^ *n* = 11.

### 3.5 Biomarker Pharmacodynamics

#### 3.5.1 sTREM2

**FIGURE 4:**
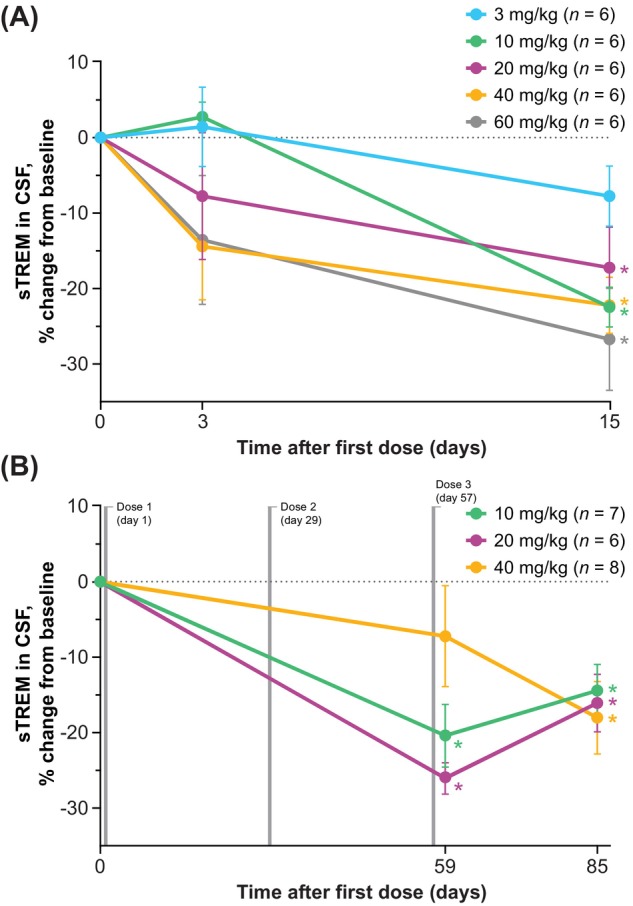
Percentage changes from baseline in sTREM2 in CSF (PD population): (A) SAD and (B) MAD. Data reported as mean ± SEM; baseline defined as predose levels. Summary data sets for these graphs are shown in Tables S2 and S3. *p < 0.05 (*t* test). CSF, cerebrospinal fluid; MAD, multiple‐ascending dose; PD, pharmacodynamic(s); SAD, single‐ascending dose; SEM, standard error of the mean; sTREM2, soluble triggering receptor expressed on myeloid cells 2.

Iluzanebart administration resulted in dose‐dependent and durable decreases in sTREM2 levels, indicating proof of target engagement (Figure 4). In the SAD CSF cohort, sTREM2 levels were markedly reduced at day 15 (14 days following dosing) with iluzanebart at doses of 10 mg/kg and above (*p* < 0.05); numerical reductions from baseline in sTREM2 levels at iluzanebart doses of 20 mg/kg and above were apparent at day 3 (2 days following dosing). In the MAD CSF cohort, a mean reduction from baseline in sTREM2 levels was observed at day 59 (2 days after the third and final dose) and was sustained through the day 85 visit (28 days after the third and final dose) in all three doses tested (*p* < 0.05).

#### 3.5.2 sCSF1R and Osteopontin/SPP1

**FIGURE 5:**
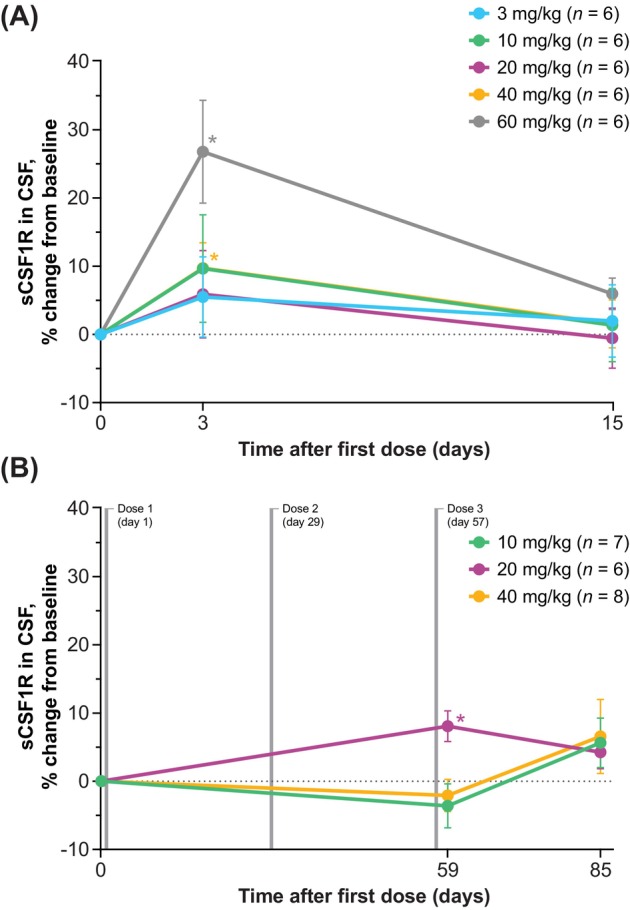
Percentage changes from baseline in sCSF1R in CSF (PD population): (A) SAD and (B) MAD. Data reported as mean ± SEM; baseline defined as predose levels. Summary data sets for these graphs are shown in Tables S2 and S3. *p < 0.05 (*t* test). CSF, cerebrospinal fluid; MAD, multiple‐ascending dose; PD, pharmacodynamic(s); SAD, single‐ascending dose; sCSF1R, soluble colony‐stimulating factor 1 receptor; SEM, standard error of the mean.

**FIGURE 6:**
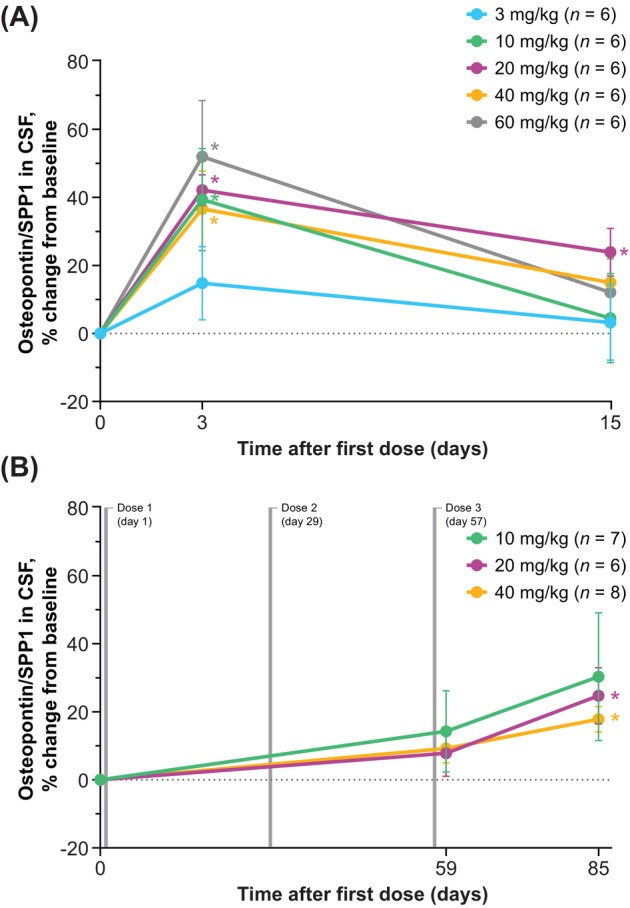
Percentage changes from baseline in osteopontin/SPP1 in CSF (PD population): (A) SAD and (B) MAD. Data reported as mean ± SEM; baseline defined as predose levels. Summary data sets for these graphs are shown in Tables S2 and S3. *p < 0.05 (*t* test). CSF, cerebrospinal fluid; MAD, multiple‐ascending dose; PD, pharmacodynamic(s); SAD, single‐ascending dose; SEM, standard error of the mean; SPP1, secreted phosphoprotein 1.

In the SAD cohorts, mean increases from baseline in the levels of microglial activity markers sCSF1R and osteopontin/SPP1 (Figure 5 and Figure 6) were observed at day 3 (2 days following the single dose). Mean increases from baseline in sCSF1R and osteopontin/SPP1 levels in the MAD cohorts were generally observed through the day 85 visit (28 days after the third and final dose).

## 4 Discussion

In this first‐in‐human, phase 1 study in HVs, TREM2 agonism with iluzanebart was safe and well tolerated at doses up to 60 mg/kg in the SAD and MAD cohorts, demonstrating a favorable safety and tolerability profile. Protocol‐specified study‐stopping criteria were not met. All investigational product–related AEs were mild or moderate in severity, and no serious AEs or clinically meaningful abnormalities in vital signs, ECGs, or laboratory parameters were reported. Across dose levels, 21.6% of HVs experienced infusion‐related reactions, which were not unexpected since these are common reactions observed with monoclonal antibody infusions [29].

Iluzanebart showed linear and predictable serum PK across doses. The observed half‐life of iluzanebart (approximately 29 days) supports monthly dosing. CNS penetration of iluzanebart (approximately 0.15% CSF‐to‐serum ratio across all doses, typical for an IgG1 monoclonal antibody) was observed. Research showing associations among neuronal injury, microglial activation, and sTREM2 shedding suggests that sTREM2 concentrations in CSF can be used as a surrogate immune biomarker of microglial activity [18, 19, 30]. Therefore, the dose‐dependent, robust, and durable reductions in CSF levels of sTREM2 shown here following single and repeat dosing with the TREM2 agonist iluzanebart demonstrate proof of target engagement, which is consistent with preclinical studies showing increased sCSF1R and decreased sTREM2 with TREM2 agonism in in vitro models of microglia under conditions of impaired CSF1R signaling [23]. Recent results from the phase 1 SAD study of AL002, an investigational monoclonal antibody against TREM2, are also directionally consistent with the data reported here in terms of target engagement and pharmacodynamic biomarker endpoints [31], although comparisons across unrelated studies should be interpreted with caution. Taken together, these data support the monthly dosing schedule of 20 mg/kg and 40 mg/kg that is being evaluated in the ongoing phase 2 trial of iluzanebart in individuals with CSF1R‐ALSP.

Similar to TREM2, CSF1R also undergoes proteolytic shedding to release its ectodomain as a soluble form (sCSF1R), and both CSF1R and TREM2 share common elements in their downstream intracellular signaling and microglial functions in the CNS following activation [1, 3, 32, 33]. Because loss‐of‐function variants in the *CSF1R* gene are the underlying cause of microglial dysfunction in CSF1R‐ALSP [1, 2], and levels of sCSF1R have been shown to be markedly reduced in carriers of *CSF1R* gene variants [32, 33], sCSF1R is also under investigation as a potential biomarker for CSF1R‐ALSP. The increases observed here in both sCSF1R and osteopontin/SPP1 levels in CSF indicate that single and repeat iluzanebart dosing had an impact on microglial activity, although the interplay between microglia and osteopontin/SPP1 in the CNS has not been fully elucidated [27, 28]. These data are consistent with the findings that preclinical data specifically from in vitro models of CSF1R‐ALSP have demonstrated that iluzanebart is a specific and potent activator of downstream human TREM2 signaling that can prevent microglial degeneration and preserve microglial viability and morphology [23, 34].

**FIGURE 7:**
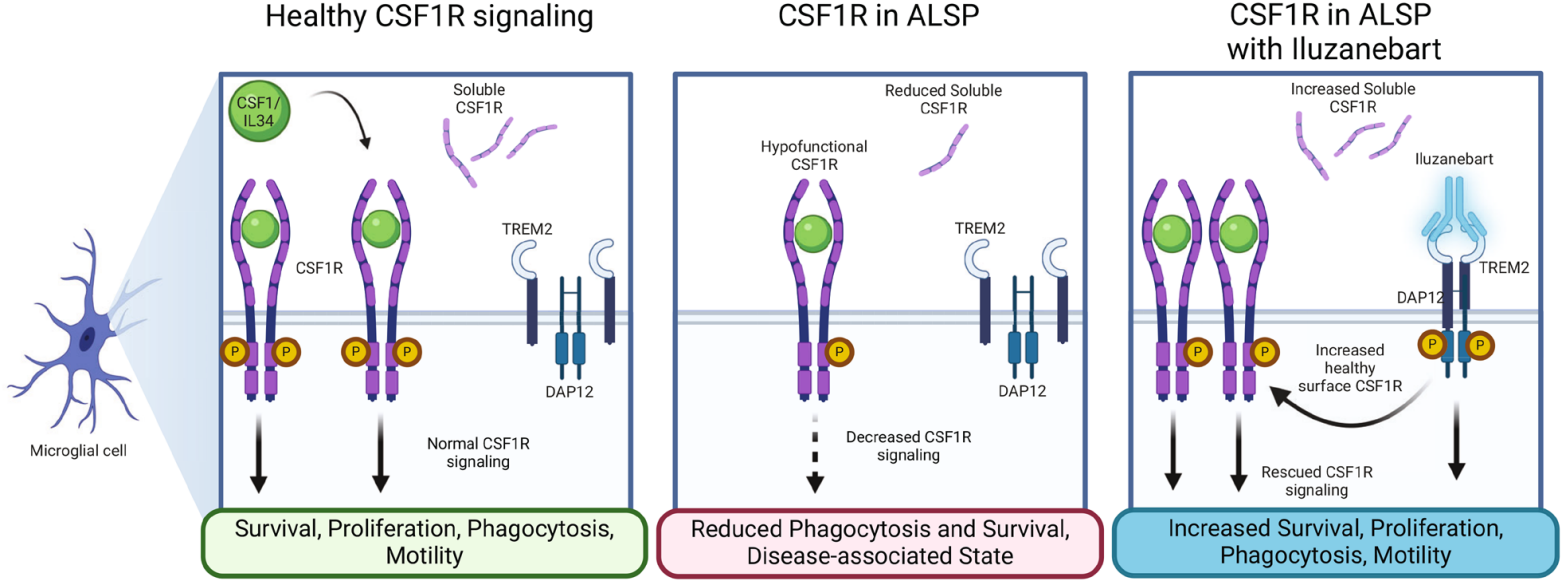
Iluzanebart proposed mechanism of action. (A) *Healthy CSF1R signaling*. Under normal conditions, ligand binding to CSF1R induces homodimerization and triggers transphosphorylation and activation of the CSF1R intracellular kinase domains, recruiting Src kinase and triggering downstream mediators such as SYK and transmembrane adapter protein DAP12 and promoting microglial survival, proliferation, phagocytosis, and motility. Ligand binding to and activation of TREM2 also trigger the same intracellular signaling pathways through pSYK and the DAP12 adapter protein. (B) *Dysfunctional CSF1R in CSF1R‐ALSP*. When CSF1R receptors are dysfunctional due to genetic mutation, signaling through microglial health‐promoting pathways is reduced. (C) *Treatment of CSF1R‐ALSP with iluzanebart*. Iluzanebart binds with high affinity to the extracellular domain of two TREM2 molecules, sequestering them in an active dimerized state and activating microglial health‐promoting downstream signaling to compensate for lost CSF1R signaling as well as augmenting signaling through an increase in the amount of CSF1 receptors at the cell surface. Reused with permission from Larson KC, et al. Rescue of in vitro models of CSF1R‐related adult‐onset leukodystrophy by iluzanebart: Mechanisms and therapeutic implications of TREM2 agonism, *J Neuroinflamm*, 22 (26), 2025, Springer Nature. ALSP, adult‐onset leukoencephalopathy with axonal spheroids and pigmented glia; CSF1, colony‐stimulating factor 1; CSF1R, colony‐stimulating factor 1 receptor; DAP12, DNAX‐activating protein of 12 kDa; IL, interleukin; SYK, spleen tyrosine kinase; TREM2, triggering receptor expressed on myeloid cells 2.

Iluzanebart represents the first antibody to demonstrate durable TREM2 engagement following repeat dosing in a clinical setting. Coupled with preclinical evidence, these phase 1 data in HVs suggest that, by activating TREM2, iluzanebart may be able to compensate for CSF1R dysfunction, thereby potentially mitigating the microglial dysfunction underpinning the neuropathological changes in CSF1R‐ALSP [32, 35]. This hypothesized mechanism of action for iluzanebart in CSF1R‐ALSP is illustrated in Figure 7 [23].

Limitations of this study include its small sample size, short duration, and population of HVs, which may preclude evaluation of some AEs and/or affect the generalizability of the results. Furthermore, efficacy assessments were not possible in this healthy population, and the open‐label nature of the CSF cohorts may have allowed for bias in some participant‐ and/or clinician‐reported assessments. Inherent to a phase 1 study in healthy participants, assessment of target engagement in this study may have been limited by a lack of impaired CSF1R signaling among the enrolled HVs. Although this study did not include or exclude enrollment based on the presence or absence of a *CSF1R* gene variant, confirmatory studies in patients with CSF1R‐ALSP are needed.

In conclusion, these findings demonstrated a favorable safety and tolerability profile for iluzanebart and confirmed iluzanebart target engagement in HVs. The safety, tolerability, PK, and PD data from this phase 1 trial support the testing of iluzanebart at doses of 20 mg/kg and 40 mg/kg in the ongoing phase 2 trial in individuals with CSF1R‐ALSP.

## Author Contributions

All authors contributed to study conception and design, data acquisition, analysis, and interpretation, and manuscript draft revisions and approved the final version of the manuscript.

## Conflicts of Interest

R.O'M. and E.A.T. are full‐time employees of Vigil Neuroscience Inc. A.Me., S.P., A.Ma., K.N., D.S., and R.R. are former full‐time employees of Vigil Neuroscience Inc. M.C. is a member of the Vigil Scientific Advisory Board and NGM Bio, is a consultant for Cell Signaling Technology, has received research grants from Vigil, NGM Bio, and Ono, and has a patent related to TREM2 pending.

## Supporting information


**Data S1.** Supporting Information.

## Data Availability

Data will be made available to qualified investigators upon reasonable request to the corresponding author.
