# Supplementary material for: Phase 1, First‐In‐Human, Single‐/Multiple‐Ascending Dose Study of Iluzanebart in Healthy Volunteers
Source: Ann Clin Transl Neurol. 2025 Apr 1;12(5):1065–76. doi: 10.1002/acn3.70033 (PMC12093347; doi:10.1002/acn3.70033)
Supplement: Supplementary file 1 — Data S1. Supporting Information. [file ACN3-12-1065-s001.docx]

**Supplemental Appendix**

**Table of Contents**

1. Inclusion criteria
2. Exclusion criteria
   1. Exclusion criteria for all participants
   2. Exclusion criteria for CSF participants
   3. Exclusion criteria for Australian participants
3. Restrictions
   1. Dietary and fluid restrictions
   2. Lifestyle considerations
   3. Concomitant medications and prohibited medications
4. Dose escalation guidelines and dose modification
5. Blinding and breaking the blind
6. Dose escalation and study stopping rules
   1. Study stopping criteria
   2. Dose escalation stopping criteria
   3. Participant discontinuation from the study
7. List of independent ethics committees (IECs) or institutional review boards (IRBs)
8. Supplemental tables and figures

Table S1. Biomarker assay performance characteristics

Table S2. Percentage changes from baseline in CSF biomarkers from SAD cohorts (PD population)

Table S3. Percentage changes from baseline in CSF biomarkers from MAD cohorts (PD population)

Figure S1. Iluzanebart pharmacokinetics (PK population): (A) SAD on day 1, (B) MAD on day 1, and (C) MAD on day 57, showing the first 2 days of each time course

**1. Inclusion criteria**

Participants who met the following criteria were considered eligible to participate in the clinical study:

1. Participant voluntarily agrees to participate in this study and signs an Institutional Review Board (IRB) approved informed consent prior to performing any of the screening visit procedures.
2. Males and females between 18 to 55 years of age, inclusive, at the screening visit.
3. All participants must conform to contraceptive requirements.
4. Participants must have a negative urine cotinine test at the screening visit and/or admission (day -2/-3).
5. Body mass index (BMI) between 18.5 and 32.0 kg/m^2^, inclusive, at the screening visit.
6. Deemed healthy by the principal investigator (PI) as determined by prestudy medical evaluation (medical history, physical examination, vital signs, 12-lead electrocardiogram [ECG], and clinical laboratory evaluations).
7. Vital signs (systolic and diastolic blood pressure and pulse rate) within acceptable ranges at screening visit and admission (day -2/-3). If values were out of range at screening visit and/or admission, 2 additional sets of vital signs were collected; if vital signs were out of range at each of these 3 consecutive assessments and were considered to be clinically relevant per investigator judgment, the participant was not eligible for study participation.

**2. Exclusion criteria**

**2.1. Exclusion criteria for all participants**

Participants who met one or more of the following criteria were not eligible to participate in the clinical study:

1. Participant had clinically significant history or evidence of cardiovascular, respiratory, hepatic, renal, gastrointestinal, endocrine, neurological, immunological, or psychiatric disorder(s) as determined by the PI or designee.
2. Participant had been administered a monoclonal antibody therapy within 120 days prior to admission (day -2/-3).
3. Participant had any concurrent disease or condition that, in the opinion of the PI, would make the participant unsuitable for participation in the clinical study.
4. Participant had history of alcohol and/or illicit drug abuse within 2 years of entry.
5. Participant had positive test for hepatitis B surface antigen (HBsAg), hepatitis C antibody, or human immunodeficiency virus (HIV) antibody.
6. Participant had positive breath test for ethanol at screening visit or admission (day ‑2/‑3).
7. Participant had positive urine drug (e.g., cocaine, amphetamines, barbiturates, opiates, benzodiazepines, and cannabinoids) and cotinine tests at screening visit or admission (day ‑2/‑3).
8. Female participants were breastfeeding or female participants with a positive serum pregnancy test at screening visit or admission (day -2/-3).
9. Participant had donated blood (>500 mL) or blood products within 2 months (56 days) prior to admission (day -2/-3).
10. Participant had been administered an investigational drug within 30 days or 5 half-lives, whichever is longer, prior to admission (day -2/-3).
11. Participant had a history of hypersensitivity to the study drug, other therapeutic monoclonal antibodies, or any of the excipients or to medicinal products with similar chemical structures.
12. Participant was unable to understand the protocol requirements, instructions and study related restrictions, the nature, scope, and possible consequences of the clinical study.
13. Participant was unlikely to comply with the protocol requirements, instructions and study related restrictions (e.g., uncooperative attitude, inability to return for follow-up visits and improbability of completing the clinical study).
14. Participant had previously been enrolled in this clinical study.
15. Vulnerable participants were defined as individuals whose willingness to volunteer in a clinical study may be unduly influenced by the expectation, whether justified or not, of benefits associated with participation, or of a retaliatory response from senior members of a hierarchy in case of refusal to participate (e.g., persons in detention, minors, and those incapable of giving consent).
16. Participant had a positive reverse transcription polymerase chain reaction (RT-PCR) test for severe acute respiratory syndrome coronavirus 2 (SARS-CoV-2) prior to admission (day -2/- 3).
17. Participant has clinical signs and symptoms consistent with SARS-CoV-2 infection (e.g., fever, dry cough, dyspnea, sore throat, fatigue, or laboratory confirmed acute infection with SARS-CoV-2).
18. Participant who had a severe course of coronavirus disease 2019 (COVID-19; i.e., had extracorporeal membrane oxygenation, mechanical ventilation, or intensive care unit stay).
19. Participant had recent (within 14 days prior to admission [day -2/-3] to the clinical unit) exposure to someone who had COVID-19 symptoms or tested positive for SARS-CoV-2.
20. Participant had recently (within 14 days prior to admission [day -2/ -3] to the clinical unit) visited a healthcare facility where COVID-19 patients were being treated.
21. Participant had received final dose of COVID-19 vaccine within 14 days prior to admission to the clinical unit (i.e., must have completed vaccination at least 14 days prior to admission).

**2.2. Exclusion criteria for CSF participants**

Participants who meet one or more of the following criteria will not be considered for inclusion in cohorts which include cerebrospinal fluid (CSF) assessments:

1. Hypersensitivity to anesthetic or derivatives used during CSF collection or any medication used to prepare the area of the lumbar puncture.
2. Previous CSF collection unrelated to this study within 30 days prior to admission (day -2/-3) to the clinical unit.
3. History of vertebral deformities, major lumbar back surgery, clinically significant back pain, or clinically significant injury that, in the opinion of the investigator, would preclude the participant from participation or CSF collection during the study.
4. An ongoing skin infection at the lumbar puncture injection site.
5. Clinically significant coagulation test values outside the normal reference range (prothrombin time/international normalized ratio, partial thromboplastin time) at screening.

**2.3. Australian-specific exclusion criteria**

1. Every day/regular smoker: an adult who has smoked at least 100 cigarettes in his or her lifetime and who now smokes every day; previously called a “regular smoker.” Someday smoker/occasional: an adult who has smoked at least 100 cigarettes in his or her lifetime and who smokes now but does not smoke every day is allowed.

**3. Restrictions**

**3.1. Dietary and fluid restrictions**

Participants should limit the consumption of coffee and caffeine-containing beverages to approximately 200 mg caffeine per day (e.g., one to two 8-ounce cups of coffee) from 48 hours prior to admission (day -2/-3) until discharge from the clinical site.

**3.2. Lifestyle considerations**

During their inpatient stay, participants must be willing to abstain from smoking (or other nicotine use) from admission (day -2/-3) until discharge from the clinical site. A participant must be willing to abstain from vigorous exercise from 48 hours prior to admission (day -2/-3) through the last inpatient stay.

**3.3. Concomitant medications and prohibited medications**

All prescription and over-the-counter medications that have been taken during the 30 days before admission (day -2/-3) through the last study visit will be recorded in the electronic case report forms (eCRFs), along with the following: reason for use, dates of administration (including start and stop dates), dose, and frequency of administration.

Any medicinal product prescribed or over-the-counter taken by a participant other than the study drug is considered concomitant medication. Permitted medication includes only contraceptives and paracetamol in recommended doses (≤2 g/day) after approval by the PI or designee. Higher doses of paracetamol (up to 3 g/day) may be given at the discretion of the PI or designee to treat adverse events such as fever and headache. Any concomitant treatment will be given only if deemed strictly necessary by the PI or designee.

Participants should refrain from the use of intravenous immunoglobulin during study participation, as this may alter the pharmacokinetics (PK) of iluzanebart.

Prohibited medications are restricted to 30 days or 5 half-lives prior to screening.

**4. Dose escalation guidelines and dose modification**

For all dose escalation cohorts, the following dose escalation procedure will be followed. The data reviewed by the Safety Review Committee (SRC) during the dose escalation meetings will be based on at least 7 days (data through day 7) of safety data following the last dose of study drug including adverse events (AEs), safety laboratory assessments, ECGs, physical examinations, neurological examinations, vital signs, and local tolerability as well as any available PK data. There should be at least 6 participants completing at least 7 days post last dose per cohort to trigger the safety review for dose-escalation; participants who discontinue or drop out may be replaced at the discretion of the Sponsor.

If consensus among the voting SRC members cannot be reached, then the PI, who has the ultimate responsibility for the safety of the volunteers, will make the final decision on the next dose level or whether to stop the study. In any event, dose escalation can only occur if agreed by the PI.

The decisions of the SRC on the next dose level will be documented and provided to all the appropriate parties involved with the study including the Pharmacist to enable study drug preparation for the next scheduled dosing day.

In case of notable AEs, safety concerns, and/or PK data during dose escalation of the study, the following changes to the next planned dose level may be considered:

- Administration of a dose below the starting dose
- Administration of an intermediate dose between the current and preceding dose
- Administration of an intermediate dose between the current and next planned dose
- Repeated administration of the current dose
- Cessation of any further dose escalation

Dose escalation will proceed until either a stopping criterion is reached or the maximum allowed exposure has been achieved.

**5. Blinding and breaking the blind**

The clinical study will be performed in a double-blind manner (Sponsor-open). All participants and site personnel (except the pharmacist on site) will be blinded to study drug assignment. The Sponsor will not be blinded to study treatment.

The study blind should not be broken except in a medical emergency (where knowledge of the study drug administered would affect the treatment of the emergency). The decision to break the blind will be made on a case-by-case basis at the discretion of the PI in collaboration with the Sponsor and/or medical monitor. The applicable standard operating procedure will be followed for blind breaking procedures.

After database lock, the overall randomization code will be broken only for reporting purposes.

Suspected unexpected serious adverse reactions that are participant to expedited reporting should be unblinded by the Sponsor before submission to the regulatory authority and the IRB.

**6. Dose escalation and study stopping rules**

Dose escalation stopping rules will be used to determine whether the maximal tolerated dose has been attained. Dose escalation may be stopped if it is determined that the limits of safety and/or tolerability have been reached. This decision will be made after a discussion takes place among voting members of the SRC. Other members of the SRC may not overrule the PI’s decision to stop dose escalation. If dose escalation is stopped due to any of the following findings, additional cohorts may receive the same or lower doses of the investigational compound.

All stopping criteria are based on U.S. Food and Drug Administration Guidance for Industry: Toxicity Grading Scale for Healthy Adult and Adolescent Volunteers (September 2007).

**6.1. Study stopping criteria**

The study will be stopped if any of the following occur:

- Two participants develop the same category Grade 3 AE related to study drug.
- One participant develops Grade 4 AE related to study drug.
- Death of a participant at any time, with relationship to study drug.

**6.2. Dose escalation stopping criteria**

Dose escalation will be stopped if any of the following occur:

- - Two or more of the participants in a cohort develop ≥ Grade 2 AEs related to study drug.
  - One or more participants in a cohort develop ≥ Grade 3 AEs.
  - It is determined that the limit of safety and/or tolerability has been reached as determined by the SRC.
  - No dose in the single- or multiple-ascending dose portion of the study will exceed 60 mg/kg. Dose escalation will be halted if PK data indicate that the predefined maximum clinical exposure level has been achieved, or is predicted to be achieved, or PK, for any reason, appear to be unpredictable. Alternatively, a dose escalation increment might be reduced so that the projected systemic exposure will not exceed predefined maximum exposure limits. The exposure limits will be applied to maximum individual values within a cohort. If any of these halting dose escalation criteria are met, the SRC will stop any further dose escalation. The SRC will determine whether a lower dose should be tested or whether the study should be terminated.
  - A dose in which the overall clinical and PK stopping criteria have been met will not be repeated and further dose escalation will not occur. Alternatively, if the clinical safety criteria support increasing the dose, a dose escalation increment might be reduced to minimize exceeding projected systemic exposure limits.
  - In some circumstances, consideration can be given to increase or reduce the infusion rate dependent on local and systemic tolerability. Any change in infusion rate needs to consider the PK exposure limits.

The infusion has to be stopped if any of the following occur:

- Grade 3 or higher infusion reaction
- Grade 2 or higher hypersensitivity reaction
- Grade 3 or higher other AE
- Breach in the vein and perivenous infusion
- Local reaction
- General reaction such as an anaphylactic-like reaction
- General bad tolerance

No dose escalation will be greater than 3-fold the previous doses administered and, for doses above 10 mg/kg, no dose escalation will be greater than 2-fold the previous doses administered.

**6.3. Participant discontinuation from the study**

Participants will be discontinued from the multiple-ascending dose part of the study if any of the following occur:

- AE ≥ Grade 3.
- Hypersensitivity or anaphylactic reaction following start of dosing.
- Medical condition that is judged by the Investigator as to jeopardize the participant’s safety if he or she continues to receive the study drug.

The stopping rules described here are applicable to staggered dosing in a cohort, dose escalation to a next cohort, and to stopping the study. Dosing may be halted temporarily to investigate before the entire study is terminated.

Measures to ensure data integrity and safety of participants include:

- Clearly defined inclusion and exclusion criteria.
- Clear individual and study stopping rules.
- Safety Review Committee for dosing decision-making; dose escalation criteria.
- International Council for Harmonisation–compliant AE monitoring, reporting and follow-up.

If the PI or designee, the medical monitor, or the Sponsor becomes aware of conditions or events that suggest a possible hazard to participants if the clinical study continues, then the clinical study may be terminated prematurely after appropriate consultation among the involved parties. The clinical study may be terminated at the Sponsor’s discretion also in the absence of such a finding.

Participant safety should take precedence over any practical, economic, or regulatory considerations.

Conditions that may warrant premature termination of the clinical study include but are not limited to:

- The discovery of an unexpected, relevant, or unacceptable risk to the participants enrolled in the clinical study.
- A decision of the Sponsor to suspend or discontinue the development of the study drug.

Should the study be terminated and/or the study center closed for whatever reason all documentation pertaining to the study and study drug must be returned to the Sponsor. Any actions of the contract research organization required for assessing or maintaining participant safety will continue as required despite termination of the study by the Sponsor.

**7. List of independent ethics committees (IECs) or institutional review boards (IRBs)**

| **Site number** | **Principal investigator and institution** | **IRB/IEC name and address** |
| --- | --- | --- |
| 001 | Hakop Gevorkyan, MD, MBA  California Clinical Trials Group  Glendale, CA, 91206, USA | WCG Institutional Review Board  1019 39th Avenue SE, Suite 120  Puyallup, WA, 98374, USA |
| 114 | Juan Carlos Rondon, MD  Clinical Pharmacology of Miami, LLC  Miami, FL, 33014, USA | Advarra Institutional Review Board  6100 Merriweather Drive, Suite 600  Columbia, Maryland, 21044, USA |
| 115 | Dr Richard Friend  Level 5 Clive Berghofer Cancer Research Centre  Herston, Queensland, Australia, 4006 | Alfred Human Research Ethics Committee  55 Commercial Road  Melbourne, Victoria, Australia, 3004 |

**8. Supplemental tables and figures**

**Table S1.** Biomarker assay performance characteristics.

|  | **sTREM2** | **sCSF1R** | **Osteopontin/SPP1** |
| --- | --- | --- | --- |
| **Platform** | R&D Systems DuoSet ELISA | R&D Systems DuoSet ELISA | Protein Simple Ella SimplePlex |
| **Matrix** | CSF | CSF | CSF |
| **Minimum required dilution** | 1/5 | 1/64 | 1/100 |
| **Calibration (quantitative range), pg/mL** | 9.96‒6080 | 62.5‒8000 | 1.34‒32,000 |
| **Buffer QC** |  |  |  |
| **Intra-assay %CV** | 5.7±3.8% | 5.9±4.9% | 2.0±1.5% |
| **Inter-assay %CV** | 8.7±3.1% | 8.4±2.4% | 5.3±0.5% |
| **Endogenous QC** |  |  |  |
| **Intra-assay %CV** | 6.7±7.4% | 4.6±5.6% | 6.0±5.5% |
| **Inter-assay %CV** | 11.4% | 16.5±2.9% | 7.7±0.6% |
| **Sample testing** | Duplicate (2 wells) | Duplicate (2 wells) | Single (1 well, 3 GNR triplicate) |
| **Study sample QC acceptance criteria** | <20% CV for duplicate (2 wells) | <20% CV for duplicate (2 wells) | <20% CV for triplicate (1 well) |

Calculations are based on study sample test reports; QCs not meeting acceptance criteria were excluded from calculations. All assays met acceptance criteria for 3 freeze-thaw cycles, demonstrated short-term stability in ambient conditions and at 2‒8 °F, and demonstrated parallelism at the minimum required dilution.

CSF, cerebrospinal fluid; CV, coefficient of variation; ELISA, enzyme-linked immunosorbent assay; GNR, glass nanoreactor; QC, quality control; sCSF1R, soluble colony-stimulating factor 1 receptor; SPP1, secreted phosphoprotein 1; sTREM2, soluble triggering receptor expressed on myeloid cells 2.

**Table S2.** Percentage changes from baseline in CSF biomarkers from SAD cohorts (PD population).

| **Change from baseline, %** | | **Iluzanebart dose level** | | | | | | | | | |
| --- | --- | --- | --- | --- | --- | --- | --- | --- | --- | --- | --- |
|  |  | **3 mg/kg** | | **10 mg/kg** | | **20 mg/kg** | | **40 mg/kg** | | **60 mg/kg** | |
|  |  | **Day 3 (*n* = 6)** | **Day 15 (*n* = 6)** | **Day 3 (*n* = 6)** | **Day 15 (*n* = 6)** | **Day 3 (*n* = 6)** | **Day 15 (*n* = 6)** | **Day 3 (*n* = 6)** | **Day 15 (*n* = 6)** | **Day 3 (*n* = 6)** | **Day 15 (*n* = 5)** |
| **sTREM2** | **Mean (SEM)** | 10.4 (5.3) | -7.8 (4.0) | 2.8 (2.0) | -22.5 (2.6) | -7.7 (8.4) | -17.2 (5.4) | -14.4 (7.1) | -22.2 (3.7) | -13.6 (8.5) | -26.7 (6.8) |
|  | **Median (range)** | -1.0 (-11.1, 25.3) | -5.0 (-27.0, -0.4) | 3.7 (-3.8, 8.3) | -23.0 (-31.5, -13.3) | -7.1 (-42.8, 14.3) | -19.1 (-35.5, 2.4) | -7.5 (-46.2, 0.1) | -22.5 (-37.4, -9.3) | -18.1 (-33.7, 17.0) | -32.3 (-38.8, -0.2) |
| **sCSF1R** | **Mean (SEM)** | 5.5 (5.9) | 2.0 (5.3) | 9.7 (7.9) | 1.4 (5.4) | 5.9 (6.4) | -0.5 (4.4) | 9.7 (3.7) | 1.5 (3.5) | 26.8 (7.5) | 1.9 (1.9) |
|  | **Median (range)** | 0.3 (-7.0, 31.0) | 3.1 (-16.6, 21.4) | 10.5 (-17.4, 38.4) | 0.6 (-20.1, 16.5) | 1.1 (-5.2, 36.4) | -2.3 (-16.4, 16.0) | 6.7 (1.7, 26.7) | 3.7 (-11.8, 9.4) | 20.3 (10.8, 55.0) | 1.8 (-20.1, 21.4) |
| **Osteopontin/ SPP1** | **Mean (SEM)** | 14.8 (10.8) | 3.2 (11.0) | 39.2 (15.0) | 4.5 (13.1) | 42.1 (4.5) | 23.9 (7.0) | 36.6 (11.2) | 15.0 (10.3) | 52.0 (16.5) | 12.1 (9.9) |
|  | **Median (range)** | 20.3(-24.7, 42.8) | 8.8 (-29.7, 31.5) | 49.3 (-22.8, 85.3) | 2.8 (-40.2, 57.6) | 39.4 (28.2, 59.3) | 24.7 (-4.0, 46.6) | 25.0 (12.7, 79.9) | 9.6 (-7.6, 60.2) | 37.7 (-24.7, 118.7) | 6.9 (-6.8, 50.3) |

Baseline defined as predose levels.

CSF, cerebrospinal fluid; PD, pharmacodynamic(s); SAD, single-ascending dose; SEM, standard error of the mean; sCSF1R, soluble colony-stimulating factor 1 receptor; SPP1, secreted phosphoprotein 1; sTREM2, soluble triggering receptor expressed on myeloid cells 2.

**Table S3.** Percentage changes from baseline in CSF biomarkers from MAD cohorts (PD population).

| **Change from baseline, %** | | **Iluzanebart dose level** | | | | | |
| --- | --- | --- | --- | --- | --- | --- | --- |
|  |  | **10 mg/kg** | | **20 mg/kg** | | **40 mg/kg** | |
|  |  | **Day 59 (*n* = 7)** | **Day 85 (*n* = 6)** | **Day 59 (*n* = 6)** | **Day 85 (*n* = 6)** | **Day 59 (*n* = 8)** | **Day 85 (*n* = 4)** |
| **sTREM2** | **Mean (SEM)** | -20.4 (4.2) | -14.4 (3.5) | -26.1 (2.1) | -16.1 (3.8) | -7.2 (6.7) | -18.0 (4.8) |
|  | **Median (range)** | -15.4 (-38.3, -‍8.9) | -13.0 (-29.5, -5.2) | -25.5 (-31.7, -21.2) | -19.7 (-26.2, -2.7) | -9.3 (-36.3, 28.8) | -20.0 (-27.4, -4.8) |
| **sCSF1R** | **Mean (SEM)** | -3.6 (3.2) | 5.6 (3.6) | 8.1 (2.3) | 4.2 (2.4) | -2.1 (2.4) | 5.3 (2.0) |
|  | **Median (range)** | -1.9 (-16.8, 5.6) | 4.8 (-6.1, 16.9) | 6.9 (1.7, 18.0) | 3.6 (-2.5, 12.7) | -2.8 (-11.6, 10.6) | 4.8 (-6.1, 19.3) |
| **Osteopontin/ SPP1** | **Mean (SEM)** | 14.2 (12.0) | 30.3 (18.8) | 7.8 (6.8) | 24.7 (8.2) | 9.2 (4.3) | 17.8 (3.7) |
|  | **Median (range)** | -0.4 (-2.9, 84.4) | 12.2 (3.1, 122.2) | 8.8 (-16.6, 28.9) | 25.6 (-5.7, 47.7) | 11.1 (-13.9, 21.9) | 15.6 (11.9, 28.1) |

Baseline defined as predose levels.

CSF, cerebrospinal fluid; MAD, multiple-ascending dose; PD, pharmacodynamic(s); SEM, standard error of the mean; sCSF1R, soluble colony-stimulating factor 1 receptor; SPP1, secreted phosphoprotein 1; sTREM2, soluble triggering receptor expressed on myeloid cells 2.

**Figure S1.** Iluzanebart pharmacokinetics (PK population): (A) SAD on day 1, (B) MAD^a^ on day 1, and (C) MAD^a^ on day 57, showing the first 2 days^b^ of each time course.^c^

**
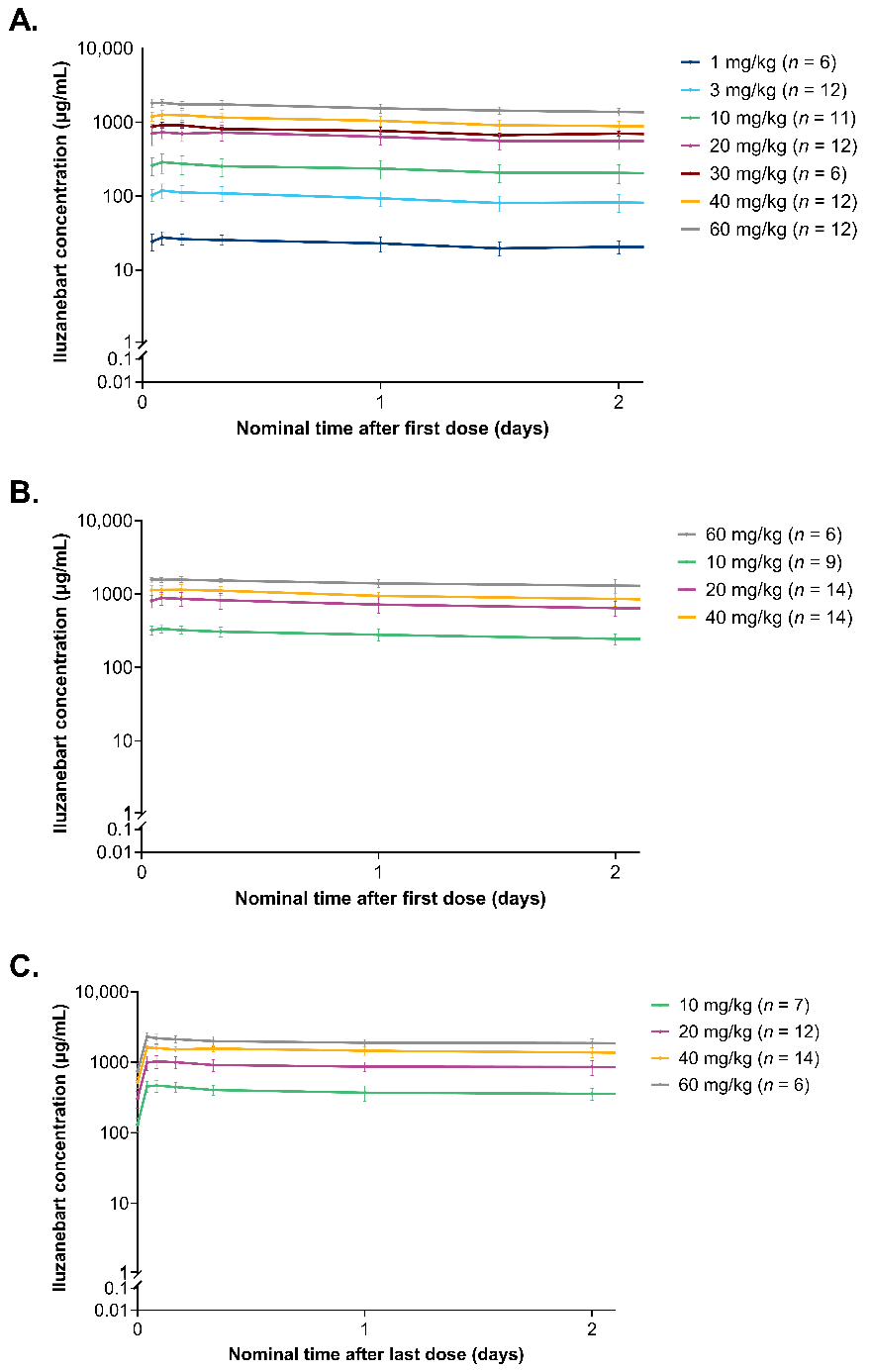
**

Data reported as mean ± SD.
^a^Three administrations at 28-day intervals.
^b^Full data out to 84 days (Panels A and C) and to 28 days (Panel B) are shown in **Figure 3** within the main text.
^c^At time zero, 1 HV in the 3 mg/kg SAD cohort and 2 HVs in the 40 mg/kg MAD cohort had non-zero concentration values (0.123–1.19 μg/mL), nominally above the quantification threshold of the assay (0.070 μg/mL) and likely due to inherent assay variability, whereas all concentrations at time zero in all other HVs were below the limit of quantification. The 3 non-zero values at time zero have therefore been excluded from the dataset as outliers.

HV, healthy volunteer; MAD, multiple-ascending dose; PK, pharmacokinetic(s); SAD, single-ascending dose; SD, standard deviation.
